# The Importance of Re-Vaccination

**Published:** 1890-02

**Authors:** 


THE IMPORTANCE OF RE-VACCINATION.—The *London Medical Recorder* quotes from a pamphlet by Dr. Gerstacker on the sanitary importance of re-vaccination as exemplified in Germany since 1874. That was the year in which the re-vaccination of school children was enforced, the result of which, according to Dr. Gerstacker, has been the almost total extinction of smallpox. From that year the German army began promptly and strikingly to feel the effect of the law, although no difference was intended or carried out in the army regulations. Dr. Gerstacker places the average duration of the protection against smallpox at ten years.—*N. Y. Medical Journal.*

